# Small-angle scattering model for efficient characterization of wood nanostructure and moisture behaviour

**DOI:** 10.1107/S1600576719002012

**Published:** 2019-03-26

**Authors:** Paavo A. Penttilä, Lauri Rautkari, Monika Österberg, Ralf Schweins

**Affiliations:** a Department of Bioproducts and Biosystems, Aalto University, Vuorimiehentie 1, 02150 Espoo, Finland; b Science Division/Large-Scale Structures Group, Institut Laue–Langevin, 71 avenue des Martyrs, 38042 Grenoble, France

**Keywords:** small-angle scattering, wood, cellulose, structural characterization, moisture behaviour

## Abstract

Small-angle scattering methods allow an efficient characterization of the hierarchical structure of wood and other cellulosic materials. However, their full utilization would require an analytical model to fit the experimental data. This contribution presents a small-angle scattering model tailored to the analysis of wood samples. The model is based on infinitely long cylinders packed in a hexagonal array with paracrystalline distortion, adapted to the particular purpose of modelling the packing of cellulose microfibrils in the secondary cell wall of wood. The new model has been validated with small-angle neutron and X-ray scattering data from real wood samples at various moisture contents. The model yields reasonable numerical values for the microfibril diameter (2.1–2.5 nm) and packing distance (4 and 3 nm in wet and dry states, respectively) and comparable results between the two methods. It is particularly applicable to wet wood samples and allows changes in the packing of cellulose microfibrils to be followed as a function of moisture content.

## Abstract 2

A small-angle scattering model for wood is presented and its capabilities for studying the nanoscale structure and moisture behaviour of wood are demonstrated.

## 1. Introduction   

Small-angle scattering of X-rays and neutrons provides a powerful tool to characterize hierarchical natural materials such as bone, collagen, silk and plant cell walls (Fratzl & Weinkamer, 2007 ▸; Müller *et al.*, 2011 ▸). In particular, the structure of the secondary cell wall of wood, which essentially consists of semi-crystalline cellulose microfibrils with a diameter of 2 to 3 nm embedded in a matrix of hemicelluloses and lignin, has been subject to numerous small-angle scattering studies (Martínez-Sanz *et al.*, 2015 ▸; Nishiyama, 2009 ▸). With these methods, the average nanoscale structure of a macroscopic sample can be observed under various conditions and with practically no need for sample preparation, which are great advantages over other techniques such as electron microscopy. However, analytical models for the interpretation of small-angle scattering data from wood and other cellulosic materials are still rare. As the quantity of scattering data produced at large-scale facilities and laboratory sources is rapidly increasing and the user community of neutrons and X-rays is ever expanding, a widely applicable and simple model for analysing small-angle scattering data from cellulosic samples would be highly desirable.

**Figure 1:**
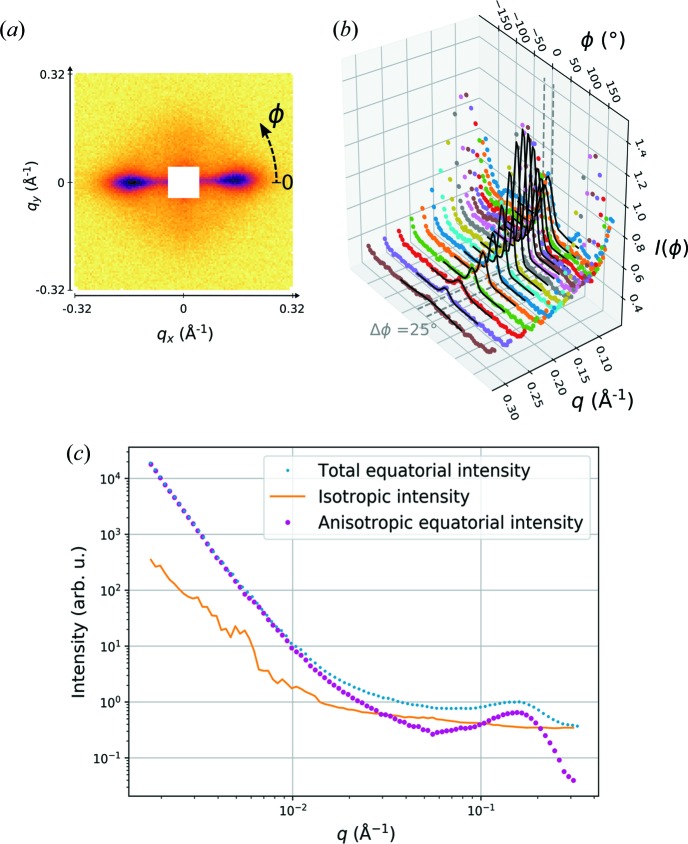
Treatment of the two-dimensional small-angle scattering data. (*a*) The SANS pattern of pine wood in D_2_O, measured at a 1.5 m sample-to-detector distance and with the fibre axis vertical. (*b*) Azimuthal intensity profiles *I*(ϕ) at different *q* values, together with the fits (solid lines) and the 25° integration sector (between dashed lines). (*c*) The effect of subtracting the isotropic intensity contribution over the complete *q* range measured with SANS.

Wood is a highly hierachical and complex material that contains a large proportion of non-cellulosic components in a composite-like structure, together with cellulose microfibrils. Nevertheless, certain features in its small-angle scattering data can be assigned to specific structural elements. The interpretation of two-dimensional scattering data from wood is largely facilitated by its strong anisotropy [Fig. 1 ▸(*a*)], which originates from the steep helical alignment of the secondary wall microfibrils around the long axis of the fibre cells (Lichtenegger *et al.*, 1999 ▸). Specific information on the cross-sectional dimensions and lateral packing of the cellulose microfibrils can therefore be extracted from the two-dimensional scattering patterns by separating the equatorial contribution from the isotropic and meridional intensity contributions, provided that a model for analysing the data exists.

Small-angle scattering data obtained from a wood sample differ slightly depending on whether neutrons or X-rays are used to collect them, leading to different approaches in the data analysis as well. Typically, equatorial small-angle X-ray scattering (SAXS) intensity profiles from wood have been analysed to obtain the cross-sectional dimensions of individual cellulose microfibrils (Jakob *et al.*, 1995 ▸), whereas small-angle neutron scattering (SANS) data have often been used to determine the packing distance between microfibrils (Fernandes *et al.*, 2011 ▸). Probably the main reason for the different approaches is that a correlation peak corresponding to the spacing between the centre points of neighbouring cellulose microfibrils (around 4 nm in wet wood) is more visible in SANS than in SAXS data (Fernandes *et al.*, 2011 ▸; Jakob *et al.*, 1996 ▸; Nishiyama *et al.*, 2014 ▸; Thomas *et al.*, 2014 ▸). However, a lack of experimental data measured with both methods from the same wood samples has made it difficult to compare data and develop a single model that could be used to extract information on both the lateral dimensions and the packing of microfibrils from experiments with either of the two methods.

The aim of the current work was to develop a model to analyse small-angle scattering data from wood samples. The model was expected to be applicable to both SANS and SAXS data from different types of wood with different moisture contents, possibly allowing automated fitting even in the hands of non-specialist users. A model based on hexagonally packed cylinders was built for this purpose, and its use is demonstrated here with SANS and SAXS data from real wood samples under various moisture conditions.

## 2. Experimental   

### 2.1. Materials   

Pieces from mature trees, including European white birch (*Betula pubescens*), Scots pine (*Pinus sylvestris*) and Norway spruce (*Picea abies*), were collected from living trees (girth 50–150 cm) in Eastern Finland and stored refrigerated in 30% ethanol solution. Radial–longitudinal (RL) and tangential–longitudinal (TL) sections of 1–2 mm thickness were cut with a knife and immersed in H_2_O for two periods of 1 to 5 days at 279 K to wash out the ethanol. The samples for the SANS experiments were subsequently immersed in D_2_O (99.9%, Sigma–Aldrich) for 1 day at around 283 K and 3 days at room temperature (about 295 K). After SANS measurements in the wet state, the samples were allowed to dry for several days under ambient conditions. For the SAXS and wide-angle X-ray scattering (WAXS) experiments in the wet state, wood sections were used either directly after washing out the ethanol solution, or after immersion in D_2_O and subsequently two periods in H_2_O. The wood samples were allowed to dry in air under ambient conditions for 6 h before the SAXS experiments in the dry state. All measurements were done for between two and seven different samples collected either from the same tree (birch and spruce) or from two individual trees (pine), and the tables of Section 3 present their mean values. The error estimates in the tables are equal to the standard deviation between samples of the same wood species and condition or the error given by the fitting software, whichever of the two was higher.

### 2.2. SANS measurements   

For the SANS measurements, the wood sections were placed in quartz cells having a light path of 2 mm, either together with excess D_2_O or in a dry state. SANS patterns were collected on the neutron instrument D11 at the Institut Laue–Langevin (ILL) (Penttilä & Schweins, 2017 ▸), using a neutron wavelength of λ = 6.0 Å (Δλ/λ = 0.09) for detector distances of 1.5, 8 and 34 m, and λ = 13 Å for a detector distance of 34 m. The total *q* range covered by this setup was from 0.0009 to 0.35 Å^−1^, with the magnitude of the scattering vector defined as *q* = 4πsinθ/λ and the scattering angle as 2θ. The two-dimensional patterns were corrected and normalized to an absolute scale using the *Large Array Manipulation Program* (*LAMP*) provided by the ILL.

### 2.3. SAXS and WAXS measurements   

SAXS and WAXS measurements were conducted on the D2AM beamline at the European Synchrotron Radiation Facility (ESRF, Grenoble, France), using an X-ray beam with wavelength λ = 0.69 Å. The SAXS regime was covered by an XPAD-D5 hybrid pixel detector set at distances of 220 and 39 cm from the sample, and the WAXS regime by a WOS detector set at a distance of 19 cm. The wet samples were measured wrapped inside a Mylar film to avoid drying and the film was cut open at the start of the SAXS measurements during drying. The temperature was maintained constant at about 295 K during the drying experiments. The two-dimensional SAXS and WAXS patterns were normalized by the transmitted beam intensity, averaged between different spots on the sample and corrected for air-scattering background.

### 2.4. Data treatment   

In order to separate the anisotropic scattering contribution from the isotropic one, the azimuthal intensity profiles from corrected and normalized two-dimensional SANS and SAXS patterns were fitted at suitably chosen *q* values with a Gaussian function around the equatorial maximum and with a linear background corresponding to the minimum of the intensity [Figs. 1 ▸(*a*) and 1 ▸(*b*)]. The minimum intensity of the azimuthal intensity profile at each value of *q* was used to approximate the isotropic scattering contribution from non-oriented components in the sample, whereas the azimuthal angle of the Gaussian peak was used to determine the centre of the equatorial integration sector for azimuthal integration. The patterns were integrated azimuthally on 25°-wide sectors around the equatorial maximum determined from SANS or SAXS data at each detector distance and the isotropic contribution was then subtracted [Fig. 1 ▸(*c*)]. For the WAXS data, the integration sectors from the low-*q* SAXS data were used. The azimuthal integration and other treatments were done with the aid of the *pyFAI* (Ashiotis *et al.*, 2015 ▸) and *FabIO* (Knudsen *et al.*, 2013 ▸) Python packages. The integrated SAXS intensities from different detector distances were merged and rebinned using the *SAXSutilities* software (http://www.sztucki.de/SAXSutilities), whereas the same procedures for the SANS intensities and the rebinning of SAXS data from drying samples were done with Python scripts.

### 2.5. Model fitting of SANS and SAXS data   

**Figure 2:**
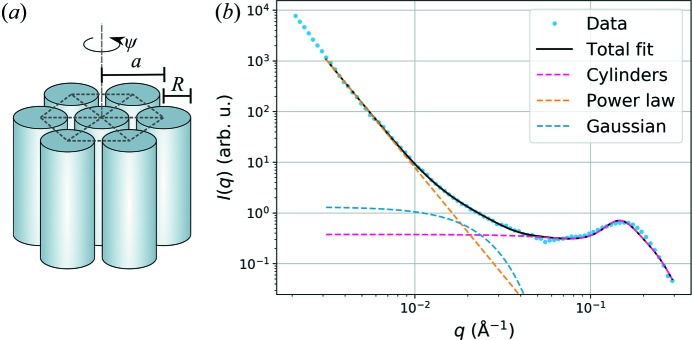
(*a*) An assembly of hexagonally packed cylinders with the definitions of radius *R*, distance *a* and azimuthal angle ψ. (*b*) An example fit to equatorial SANS data from pine wood in D_2_O, showing the different contributions of equation (1).

The equatorial small-angle scattering intensity from wood samples was fitted with the function 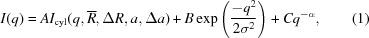
where *A*, *B*, σ, *C* and α are constants and *I*_cyl_(*q*) is the intensity from infinitely long cylinders organized in a hexagonal lattice with paracrystalline lattice distortion of the second kind (Hosemann & Bagchi, 1962 ▸), based on the work of Hashimoto *et al.* (1994 ▸) ▸ (Appendix *A*1) and modified at low *q* as detailed in Appendix *A*2. The paracrystalline distortion of the distance *a* between the cylinders’ centre points is characterized by Δ*a* and the polydispersity of the cylinder radius *R* by a Gaussian distribution with mean 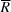 and standard deviation Δ*R* [Fig. 2 ▸(*a*)]. The rotation of the crystals around the cylinder axis is assumed to be uniform [the angle ψ in Fig. 2 ▸(*a*) is random].

In the model of equation (1), the cylinders are assumed to correspond to the cellulose microfibrils in the S_2_ layer of wood’s secondary cell wall. This particular analytical model was chosen because it provides a simple way of describing the average cross-sectional dimensions and packing distance of the microfibrils with polydispersity in both. From the other terms present in equation (1), the power-law scattering term (term with *C*) at low *q* with α close to 4 is assigned to the surfaces of larger pores and fibre lumina (Jakob *et al.*, 1996 ▸; Nishiyama *et al.*, 2014 ▸), whereas the Gaussian function centred at *q* = 0 Å^−1^ (term with constant *B*) was added to approximate scattering from larger pores or other unspecified structural features and to reach a good fit for all data except the SAXS data from wet softwoods. The relative contributions of the different terms in a fit to a real wood sample are illustrated in Fig. 2 ▸(*b*).

The model fitting of equatorial SAXS and SANS intensities of wet and dry samples, after subtracting the isotropic component as described in Section 2.4, was done in the *SasView 4.1.0* software (Doucet *et al.*, 2017 ▸) using a plugin written in Python 2, whereas the SAXS data measured during drying were fitted using customized Python scripts. An error of 2% for the intensity data points was used to weight the fits in all cases. The Gaussian distribution of the cylinder radius [equation (8)] was computed with 11 points between 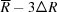 (or 0 if < 0) and 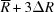, and the orientational average [equation (3)] with 1001 values between 0 and 2π for ψ.

### 2.6. Determination of crystallite dimensions from WAXS data   

Crystal size was calculated from equatorial WAXS data from the dry wood samples. The intensity between *q* values of 0.5 and 2.25 Å^−1^ was fitted with three Gaussian functions corresponding to the reflections *hkl* = 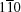, 110 and 200 of cellulose I_β_ (Nishiyama *et al.*, 2002 ▸), and one broad Gaussian function centred around *q* = 1.4 Å^−1^ plus a linear function corresponding to the amorphous background and other reflections. The crystal size *L_hkl_* in each of the three directions was calculated using the Scherrer equation: 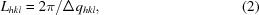
where Δ*q_hkl_* is the integral breadth of the diffraction peak. The values of *L_hkl_* are often regarded as lower limits for the true crystal size, because other factors such as lattice distortions also contribute to peak broadening (Fink *et al.*, 1995 ▸). On the other hand, in the case of cellulose the crystal size could be overestimated owing to the irregular structure of the microfibrils, including a size distribution of the diameter and possible joins between neighbouring microfibrils (Leppänen *et al.*, 2009 ▸).

## 3. Results and discussion   

### 3.1. Small-angle scattering results   

#### 3.1.1. SANS results from wet and dry wood samples   

**Figure 3:**
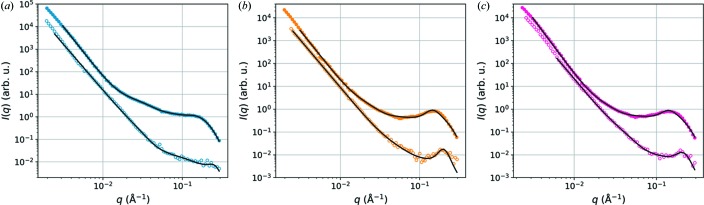
Example SANS data from wood samples under wet (filled symbols) and dry (open symbols) conditions, with the fits shown with continuous lines: (*a*) birch, (*b*) pine and (*c*) spruce.

Example fits of the model [equation (1)] to equatorial SANS data from wet and dry wood samples are presented in Fig. 3 ▸ and the fitting results in Table 1 ▸. Owing to the limited *q* range and the weakness of the shoulder feature related to the form factor of the cellulose microfibrils, the relative standard deviation of the mean radius 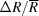 was fixed to 0.2 in all fits and the mean radius 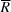 to 1.0 nm in the fits to the data from dry samples. Even then, the correlation peak related to fibrillar packing in the SANS data from dry birch samples was too weak to be observed or reliably fitted, and the values of *a* and Δ*a* for dry birch in Table 1 ▸ should be considered only as rough estimates.

As shown by Fig. 3 ▸, the model fits well to the equatorial SANS data from all wet samples and reproduces the correlation peak around *q* = 0.15 Å^−1^ with reasonable values for the interfibrillar distance *a* (Table 1 ▸). However, the contribution modelled by the Gaussian term of the model [term with *B* in equation (1)] is particularly strong in the wet birch samples (*q* range from 0.02 to 0.07 Å^−1^), disturbing slightly the determination of the distance *a* and leading to less reliable results than in the softwood samples.

As a result of drying, the low-*q* power law with exponent α close to 4 extends to higher *q* values, accompanied by a weakening and shift of the correlation peak from around *q* = 0.15 Å^−1^ (real-space distance *d* = 2π/*q* = 4.2 nm) almost up to *q* = 0.2 Å^−1^ (*d* = 3 nm) in samples where it is still visible. The shift and weakening of the correlation peak in SANS data from softwoods have previously been explained by a closer packing of the cellulose microfibrils as water is removed from spaces between them (Fernandes *et al.*, 2011 ▸; Plaza *et al.*, 2016 ▸). This change in structure is also well reflected in the value of *a*, which decreases from around 4.2 nm in wet softwoods to around 3 nm in the corresponding dry samples (Table 1 ▸). The diameter of the cellulose microfibrils 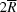 varies around 2.1 nm in all wet wood samples.

#### 3.1.2. SAXS results from wet and dry wood samples   

**Figure 4:**
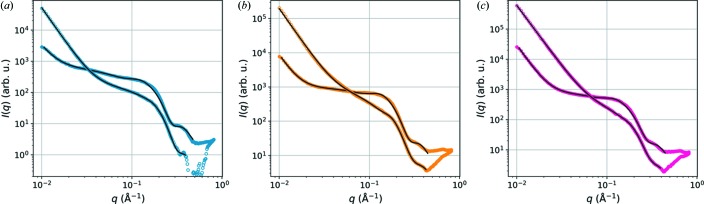
Example SAXS data from wood samples under wet (filled symbols) and dry (open symbols) conditions, with the fits shown with continuous lines: (*a*) birch, (*b*) pine and (*c*) spruce.

Example fits of the model [equation (1)] to equatorial SAXS data from wet and dry wood samples are presented in Fig. 4 ▸ and the fitting results in Table 2 ▸. The cylinder term (term with *A*) of equation (1) dominates the fits above *q* = 0.1 Å^−1^, where the SAXS intensity mostly arises from the lateral cross section of the cellulose microfibrils (Jakob *et al.*, 1995 ▸). With the assumption of a circular microfibril cross section, mean diameters (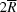) from 2.4 to 2.5 nm with 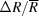 about 0.2 are obtained for all samples in the wet state. In the dry samples, the microfibrils appear around 10% thinner, with values of 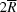 between 2.1 and 2.2 nm (Table 2 ▸).

In addition to the microfibril cross section, the shape of the shoulder feature between 0.1 and 0.3 Å^−1^ carries information on the lateral packing of the cellulose microfibrils (Jakob *et al.*, 1996 ▸; Leppänen *et al.*, 2009 ▸), though less distinctively than the 0.15 Å^−1^ peak in the SANS data (Section 3.1.1). The current model enables the determination of an average packing distance *a* for the cellulose microfibril cross sections from the SAXS data from both wet and dry samples and the observation of its decrease from around 4 nm to close to 3 nm as a consequence of drying (Table 2 ▸). Moreover, the equatorial SAXS intensities exhibit a similar weakening of the shoulder feature and strengthening of the power-law scattering at low *q* to that seen with the SANS data (Section 3.1.1), except that the onset of the power-law scattering is shifted even more clearly from about 0.03 Å^−1^ (20 nm) to about 0.06 Å^−1^ (10 nm) as a result of drying. The strong increase in the power-law scattering with almost unchanged exponent α ≃ 4 has previously been explained by the increased contrast between larger pores and the cell wall when water is gradually exchanged for air in the pores and voids of the wood tissue (Jakob *et al.*, 1996 ▸; Leppänen *et al.*, 2011 ▸; Suzuki & Kamiyama, 2004 ▸).

#### 3.1.3. SAXS results from drying wood samples   

**Figure 5:**
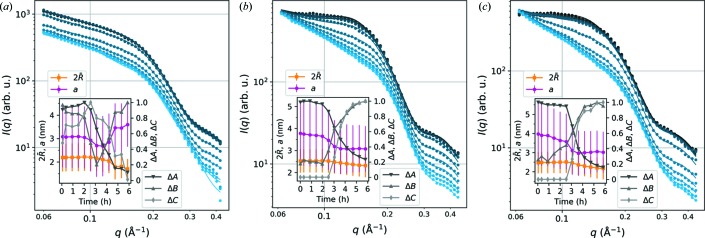
SAXS data measured during drying of wood samples (from dark to light colour), with the fits shown with continuous lines: (*a*) birch, (*b*) pine and (*c*) spruce. The insets present the change in fitting parameters 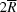 and *a* (*y* axis on the left), with the fitting parameters 2Δ*R* and Δ*a* illustrated by error bars, and the change in *A*, *B* and *C* relative to their maximum values (*y* axis on the right).

In order to examine the gradual changes in the SAXS data with decreasing moisture content, the wood samples were allowed to dry in the sample holder and SAXS patterns at the shorter sample-to-detector distance (39 cm) were measured at about 30 min time intervals during drying. The resulting equatorial SAXS intensities, shown in Fig. 5 ▸, were fitted with the model of equation (1). Owing to the limited *q* range, the power-law exponent α was fixed to 4 and the upper boundary of σ to 0.08 in all fits. The insets of Fig. 5 ▸ show the values of the mean microfibril diameter (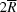) and the interfibrillar distance (*a*), with the polydispersities 2Δ*R* and Δ*a* indicated by error bars. Also shown are the time-dependent changes in the constant factors *A*, *B* and *C* relative to their maximum values. The value of σ remains almost constant between 0.073 and 0.08 in all samples. Unfortunately, the fits to the third and fourth last time points of the birch sample [Fig. 5 ▸(*a*)] led to unstable fits and unrealistic fitting parameters because of the limited *q* range, and will therefore be excluded from the following discussion.

The SAXS intensities of the drying wood samples follow the general trends of a weakening shoulder feature (parameter *A*) and increasing contribution of the low-*q* power-law scattering (*C*) (Fig. 5 ▸). Furthermore, the interfibrillar distance *a* decreases by about 20% in the softwood samples and by 10% in the birch sample (the first eight time points). Also, the microfibril diameter (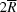) decreases during drying in all samples. In most cases, substantial changes in the SAXS data and sample structure take place within a relatively short time interval, which can be seen as a sudden change in the fitting parameters around 3 h drying time. Moreover, the nanoscale structure, at least in the softwood samples, seems to reach an equlibrium during the course of the 6 h experiment, as indicated by the levelling off of the values at the end of the experiment. The exact time dependence and rate of structural changes vary between samples because of differences in the shape and thickness of the wood samples and the uncontrolled environment. However, the current data demonstrate the capabilities of the model of equation (1) to quantify moisture-dependent changes in the wood nanostructure and to result in reasonable outcomes, even from SAXS data measured over a limited *q* range.

### 3.2. WAXS results   

**Figure 6:**
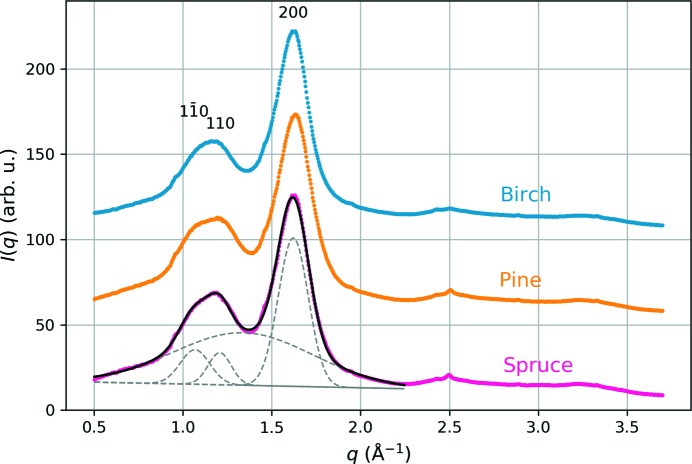
Representative equatorial WAXS data from dry wood samples, with an example fit to the data from spruce wood.

WAXS data were measured in order to obtain an independent estimate for the lateral dimensions of the cellulose microfibrils in the wood samples. Representative equatorial WAXS intensities of dry wood samples with an example fit are presented in Fig. 6 ▸. The lateral crystallite dimensions obtained by peak fitting (Table 3 ▸) correspond to a roughly circular or elliptic cross section of the cellulose microfibril in all samples. The diameter of the microfibril cross section, averaged over the different lateral directions, is between 3.2 and 3.6 nm in all samples, with a slightly larger value for birch than the softwoods. However, the crystallite thickness in the direction perpendicular to the plane of the glucose units, calculated from the best-resolved 200 reflection, is close to 3.0 nm in all samples.

### 3.3. Discussion   

Despite the relatively long history of SAXS and SANS studies of wood and other cellulosic fibres, there is no clear consensus on the interpretation of the experimental scattering data. SAXS data from wood have often been fitted over a rather limited *q* range, using a cylinder form factor or a simple Guinier law for the cellulose microfibrils (Cheng *et al.*, 2011 ▸; Guo *et al.*, 2016 ▸; Jakob *et al.*, 1995 ▸; Jungnikl *et al.*, 2008 ▸; Smith *et al.*, 2012 ▸; Suzuki & Kamiyama, 2004 ▸) and also considering sometimes their packing into bundles (Barbetta *et al.*, 2017 ▸; Jakob *et al.*, 1996 ▸; Leppänen *et al.*, 2009 ▸). SANS data, on the other hand, are most typically analysed by extracting the location of the interfibrillar correlation peak observed around *q* = 0.15 Å^−1^ (Fernandes *et al.*, 2011 ▸; Nishiyama *et al.*, 2014 ▸; Plaza *et al.*, 2016 ▸; Thomas *et al.*, 2014 ▸), thereby neglecting any information on the cross-sectional size of the individual cellulose microfibrils. In this work, a model that can be used to analyse both SANS and SAXS data from wood samples at various moisture contents and over a wide *q* range has been presented. Fitting of the full model requires the optimization of nine parameters, but the task is facilitated by the separate *q* ranges where the different contributions dominate [Fig. 2 ▸(*b*)]. The full model covers the structure of the secondary cell wall of wood, from the level of microfibrils to the surface of the cell lumina.

Fits of the model [equation (1)] to SANS and SAXS data from wet and dry wood samples yielded values for the mean diameter of the cellulose microfibrils (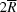 in Tables 1 and 2) and their packing distance (*a* in Tables 1 and 2) that are well in line with each other and with previous results found in the literature, as will be discussed in the following. In particular, the SAXS results for the microfibril diameter in wet pine and spruce (2.5 nm with about 20% polydispersity) are in excellent agreement with the thorough analysis by Jakob *et al.* (1995 ▸) ▸, which resulted in a microfibril diameter of 2.5 nm with less than 10% polydispersity in spruce wood. The corresponding values for wet birch wood and for the dry samples were slightly smaller, but still close to the range of 2.4–2.7 nm determined with SAXS for various woods (Guo *et al.*, 2016 ▸; Leppänen *et al.*, 2009 ▸, 2011 ▸; Suzuki & Kamiyama, 2004 ▸). The values of 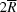 obtained from fits to the SANS data from wood samples in D_2_O were about 0.5 nm smaller than those from the SAXS fits, which could be explained by H/D exchange between the solvent and the outermost layer of cellulose molecules on the microfibril surface. The microfibril diameters from the SAXS fits (Table 2 ▸) were smaller than the crystal thickness determined from the best-resolved 200 reflection in the WAXS intensities (Table 3 ▸), which is an issue that has also been encountered by other authors (Leppänen *et al.*, 2009 ▸) and which could be explained by the approximate nature of the Scherrer equation, especially when applied to cellulosic samples (as discussed in Section 2.6).

Besides the microfibril diameter, the model was able to determine the interfibrillar distance *a* from both SANS and SAXS data. The values of *a* based on SANS data from the softwoods under wet and dry conditions (Table 1 ▸) were similar to those obtained previously by simpler fits (Fernandes *et al.*, 2011 ▸; Plaza *et al.*, 2016 ▸) and in good agreement with those estimated by Bertinetti *et al.* (2016 ▸) ▸ using a geometric model for the microfibril packing at different moisture contents. Additionally, the current model yielded realistic estimates for the polydispersity of *a* (Δ*a*/*a* from 0.2 to 0.3). For some reason, the value of *a* determined from the SANS data from wet birch wood was slightly smaller than those from the softwoods, but this is in line with the results of Thomas *et al.* (2014 ▸) ▸. The values of *a* obtained from the SAXS fits (Table 2 ▸) were similar to previous results determined with the same method but a slightly different model (Leppänen *et al.*, 2009 ▸, 2011 ▸), and they reflected similar changes in the SANS data during drying. However, the values of *a* based on SAXS fits were slightly smaller than those based on SANS data, which might be due to the more difficult resolution of the interfibrillar correlation peak in the SAXS data. The intrinsic differences between SANS and SAXS data obtained from the same wood sample are probably related to the different scattering length density contrasts seen by the two probes, which make them sensitive to slightly different structures depending on the chemical or isotopic composition and the local distribution of the different components in the system.

In order to demonstrate the performance of the model further, drying of wood samples was monitored with *in situ* SAXS measurements. The fits to the data (Fig. 5 ▸) were able to capture behaviour in the nanoscale structure of the wood cell wall similar to what has been reported before, most importantly the gradual change in the spacing between the microfibrils with changing moisture content (Fernandes *et al.*, 2011 ▸; Leppänen *et al.*, 2011 ▸; Plaza *et al.*, 2016 ▸). A decrease in the value of *a* during drying was observed in all samples, but the change was less drastic in birch. The different behaviour of birch wood might be explained by its originally lower value of *a* compared to the softwood samples. Previously, Thomas *et al.* (2014 ▸) ▸ reported no effect of moisture content on the position of the correlation peak in SANS data from birch, whereas a roughly 10% decrease in the interfibrillar distance was reported by Leppänen *et al.* (2011 ▸) ▸ on the basis of SAXS data measured during drying. Another interesting point is the decrease in the microfibril diameter with decreasing moisture content, which was observed consistently in the SAXS data from all samples. A similar phenomenon can be seen in the results of some previous SAXS studies (Cheng *et al.*, 2011 ▸; Suzuki & Kamiyama, 2004 ▸), as well as in reports of decreasing lateral crystal size based on WAXS data from drying wood samples (Leppänen *et al.*, 2011 ▸; Toba *et al.*, 2013 ▸; Yamamoto *et al.*, 2010 ▸). Such WAXS results might indicate a connection with mechanical stresses caused by moisture changes, but the subject deserves further study.

The current SANS and SAXS data analysis reveals some differences in the nanoscale structure of samples from different wood species and how they respond to moisture changes. The results show a shorter interfibrillar distance *a* for birch than for pine or spruce, which would indicate a tighter packing of the cellulose microfibrils in birch compared with the two softwoods. This observation is in line with previous SANS results from spruce (Fernandes *et al.*, 2011 ▸) and birch (Thomas *et al.*, 2014 ▸), and offers an explanation for the poorer resolvability of the 0.15 Å^−1^ correlation peak and the relatively smaller decrease in the distance *a* during drying of birch wood. Also, the stronger presence of the scattering contribution in the *q* range from roughly 0.02 to 0.07 Å^−1^, modelled by the Gaussian function centred at *q* = 0 Å^−1^, seems to be more characteristic of the birch sample than the two other woods. Interestingly, similar intensity contributions have also been observed in SANS data from poplar wood (Sawada *et al.*, 2018 ▸) and in SAXS data from delignified spruce wood (Jungnikl *et al.*, 2008 ▸), both of which were thought to be related to nanoscale pores located between small bundles of cellulose microfibrils. The origin of this scattering feature and the detailed differences between the scattering patterns from hardwoods and softwoods are beyond the scope of this article but will be addressed in future work.

## 4. Conclusions   

A model for analysing the lateral dimensions and packing distance of cellulose microfibrils from small-angle scattering data from wood samples has been constructed. The model’s capabilities have been demonstrated with SANS and SAXS data from wood samples under various moisture conditions and the results are consistent with each other and the existing literature. The obtained small-angle scattering data and fitting results indicate structural differences between hardwoods and softwoods, which slightly affect the applicability of the model and which should be studied further in order to better understand the origin of different features in small-angle scattering data from wood samples. Also, the response of the wood cell wall to moisture changes on the nanometre level should be explored using better controlled humidity conditions and complementary experimental methods. Results from such experiments will be reported in the near future.

The model of equation (1) will be made publicly available as a plugin (*WoodSAS*) to the *SasView* fitting software at the SasView Marketplace (http://marketplace.sasview.org/).

## Figures and Tables

**Figure 7:**
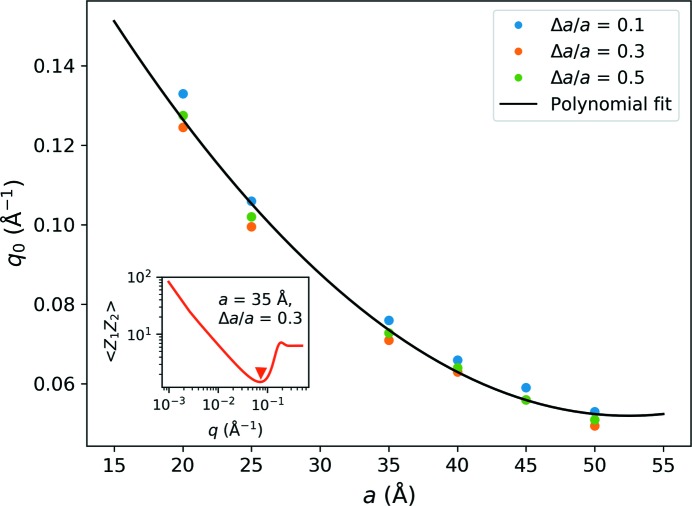
Determination of the turning point *q*_0_ to be used in equation (13), based on a polynomial fit to the observed locations of the first minimum of azimuthally averaged *Z*_1_ *Z*_2_ (indicated by a triangle in the example shown in the inset).

**Figure 8:**
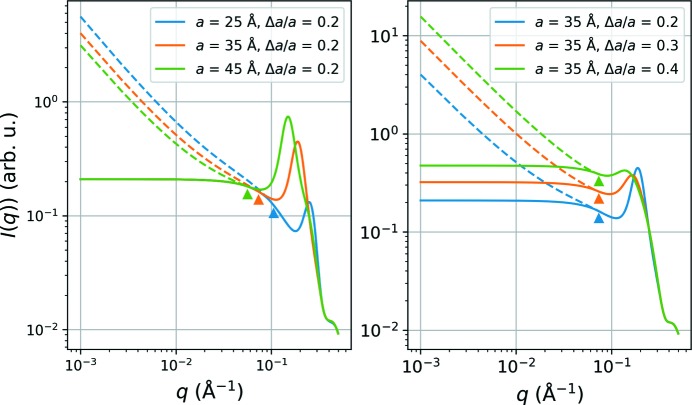
The effects of modifying the lattice factor *Z*(*q*) according to equations (13) and (14) (with 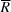 = 10 Å and 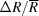 = 0.2), with the solid and dashed lines corresponding to the modified and unmodified expressions, respectively, and triangles marking the location of the turning point *q*_0_.

**Table 1:** Results of fits to equatorial SANS intensities from wet and dry wood samples, with fixed parameters marked with asterisks

Sample	*A*	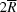 (nm)	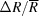	*a* (nm)	Δ*a*/*a*	*B*	σ (× 10^−2^)	*C* (× 10^−7^)	α
Birch, wet	3.2 ± 0.5	2.16 ± 0.05	0.2*	3.1 ± 0.2	0.46 ± 0.05	6.4 ± 0.5	2.2 ± 0.1	0.7 ± 0.4	4.4 ± 0.2
Birch, dry	0.09 ± 0.03	2.0*	0.2*	2.27 ± 0.09	0.26 ± 0.02	0.6 ± 0.3	1.2 ± 0.2	1.2 ± 0.5	4.02 ± 0.04
Pine, wet	1.7 ± 0.4	2.04 ± 0.02	0.2*	4.21 ± 0.04	0.271 ± 0.006	2.4 ± 0.9	1.48 ± 0.04	0.31 ± 0.07	4.34 ± 0.06
Pine, dry	0.03 ± 0.01	2.0*	0.2*	3.3 ± 0.2	0.21 ± 0.03	0.03 ± 0.01	3.6 ± 0.7	0.4 ± 0.2	4.13 ± 0.02
Spruce, wet	1.6 ± 0.3	2.07 ± 0.01	0.2*	4.23 ± 0.02	0.31 ± 0.01	2.5 ± 0.2	1.51 ± 0.03	0.08 ± 0.02	4.8 ± 0.2
Spruce, dry	0.04 ± 0.01	2.0*	0.2*	2.91 ± 0.05	0.23 ± 0.03	0.09 ± 0.05	3.4 ± 0.9	0.5 ± 0.1	4.21 ± 0.07

**Table 2:** Results of fits to equatorial SAXS intensities from wet and dry wood samples

Sample	*A* (× 10^3^)	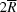 (nm)	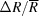	*a* (nm)	Δ*a*/*a*	*B* (× 10^3^)	σ (× 10^−2^)	*C* (× 10^−3^)	α
Birch, wet	1.0 ± 0.3	2.4 ± 0.2	0.23 ± 0.03	3.74 ± 0.04	0.51 ± 0.04	0.7 ± 0.5	3.2 ± 0.3	0.2 ± 0.1	3.86 ± 0.08
Birch, dry	0.16 ± 0.08	2.2 ± 0.2	0.21 ± 0.02	3.19 ± 0.06	0.38 ± 0.04	0.3 ± 0.1	8.7 ± 0.5	0.7 ± 0.4	4.10 ± 0.01
Pine, wet	1.5 ± 0.4	2.51 ± 0.02	0.207 ± 0.003	3.75 ± 0.05	0.41 ± 0.01			0.04 ± 0.03	4.2 ± 0.2
Pine, dry	0.3 ± 0.1	2.19 ± 0.07	0.24 ± 0.01	3.31 ± 0.06	0.300 ± 0.008	0.47 ± 0.09	7.4 ± 0.2	4 ± 1	3.76 ± 0.02
Spruce, wet	1.0 ± 0.3	2.46 ± 0.02	0.222 ± 0.002	4.1 ± 0.2	0.460 ± 0.003			0.04 ± 0.04	4.5 ± 0.2
Spruce, dry	0.13 ± 0.06	2.1 ± 0.1	0.253 ± 0.005	3.2 ± 0.1	0.35 ± 0.01	0.25 ± 0.03	8.0 ± 0.3	4 ± 2	3.98 ± 0.04

**Table 3:** Lattice spacings (*d_hkl_*) and crystal dimensions (*L_hkl_*) obtained from fits to equatorial WAXS data from dry wood samples

Sample	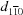 (nm)	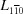 (nm)	*d*_110_ (nm)	*L*_110_ (nm)	*d*_200_ (nm)	*L*_200_ (nm)
Birch	0.5780 ± 0.0007	2.95 ± 0.04	0.5211 ± 0.0004	4.73 ± 0.09	0.3891 ± 0.0009	3.09 ± 0.02
Pine	0.590 ± 0.004	3.0 ± 0.1	0.516 ± 0.002	3.8 ± 0.1	0.3860 ± 0.0006	2.86 ± 0.02
Spruce	0.588 ± 0.001	2.98 ± 0.03	0.518 ± 0.002	4.0 ± 0.1	0.3868 ± 0.0009	2.956 ± 0.004

## Appendix A Detailed description of the small-angle scattering model

### A1. Scattering from a hexagonal array of cylinders

According to Hashimoto *et al.* (1994 ▸) ▸, the equatorial intensity measured from infinitely long cylinders (radius *R*) organized in a hexagonal lattice (lattice constant *a*) with paracrystalline lattice distortion of the second kind (Hosemann & Bagchi, 1962 ▸) and random orientation of the crystals around the cylinder axis [Fig. 2 ▸(*a*)] is

where

The form factor of an infinitely long cylinder in equation (4) is

with *J*_1_ denoting the Bessel function of the first kind and *A*_cyl_ the cross-sectional area of the cylinder.

The terms with averaging in equation (4) are

and

where *P*(*R*) is the Gaussian distribution of a cylinder of radius *R*:

The paracrystal lattice factors *Z*_1_ and *Z*_2_ for a hexagonal lattice with lattice vectors **a**_1_ and **a**_2_ are obtained from

where

### A2. Modified lattice factor of a hexagonal array of cylinders

In the model of Appendix *A*1, the intensity presented in equation (3) tends to increase as *q* approaches zero. This behaviour is caused by the lattice factor *Z*_*k*_(*q*) [equation (9)] and is related to the random azimuthal orientation of the infinitely large paracrystals (Matsuoka *et al.*, 1987 ▸), making the theory incapable of fully representing the scattering from finite complex structures such as the organization of microfibrils in the wood cell wall. In order to avoid the upturn in intensity at low *q*, the value of *Z_k_* in equation (4) is approximated by the following expression:

The value of the turning point *q*_0_ in equation (13) is chosen to correspond to the first minimum in *Z*_1_ *Z*_2_ calculated from equation (9) and averaged over all angles ψ as in equation (3) (marked with a triangle in the inset of Fig. 7 ▸). In the relevant parameter range, the value of *q*_0_ (in Å^−1^) depends only on the lattice spacing *a* (in ångströms) and is approximated by the polynomial

as presented in Fig. 7 ▸. The effect of this modification on the orientationally averaged intensity with different values of *a* and Δ*a* is illustrated in Fig. 8 ▸.
